# Combining Ability of *Capsicum annuum* Hybrid for Antioxidant Activities, Polyphenol Content, α-Glucosidase Inhibitory, Yield, and Yield Components

**DOI:** 10.3390/cimb46100695

**Published:** 2024-10-21

**Authors:** Muhamad Syukur, Awang Maharijaya, Waras Nurcholis, Arya Widura Ritonga, Arya Yuda Pangestu, Andi Nadia Nurul Lathifa Hatta, Muhammad Ridha Alfarabi Istiqlal, Abdul Hakim, Zulfikar Damaralam Sahid

**Affiliations:** 1 Department of Agronomy and Horticulture, Faculty of Agriculture, IPB University, Jl. Meranti, IPB Dramaga Campus, Bogor 16680, Indonesia; awangmaharijaya@apps.ipb.ac.id (A.M.);; 2 Center for Tropical Horticulture Studies, IPB University, Jl, Raya Padjajaran, IPB Baranangsiang Campus, Bogor 16680, Indonesia; 3 Department of Biochemistry, Faculty of Mathematics and Natural Sciences, IPB University, Jl, Agatis, IPB Dramaga Campus, Bogor 16680, Indonesia; wnurcholis@apps.ipb.ac.id; 4 Research Center for Genetic Engineering, Research Organization for Life Sciences and Environment, National Research and Innovation Agency, Bogor 16915, Indonesia; andinadiasp@gmail.com; 5 Study Program of Agrotechnology, Faculty of Industrial Technology, Gunadarma University, Jl, Margonda Raya, Depok 16451, Indonesia; 6 Study Program of Agrotechnology, Faculty of Agriculture, Siliwangi University, Jl, Peta, Tasikmalaya 46196, Indonesia; 7 Vocational School of Sciences, IPB University, Jl, Kumbang, Bogor 16680, Indonesia

**Keywords:** α-glucosidase inhibition, antioxidant, combining ability, heterotic effect, polyphenol content

## Abstract

Chili (*Capsicum annuum*) consumption is often suggested, and using functional food cultivars is the most effective strategy post COVID-19 pandemic. Controlling chili breeding activity is one of the most effective methods to produce new hybrid varieties. However, the general combining ability (GCA), specific combining ability (SCA), and heterotic effect of functional biochemicals (polyphenol content, antioxidant activities, and α-glucosidase inhibitory compounds) remain poorly known in *C. annuum*. This study aimed to estimate these parameters in *C. annuum* by using five different genotypes and their hybrid combinations based on growth characteristics, yield, yield components, and fruit functional biochemicals. The F1 and F1R progenies were obtained from crosses in a greenhouse with a full diallel mating design. Each parent used in this study had a GCA advantage for each characteristic. The hybrid combination of IPB074 × IPB005 and IPB435 × IPB367 displayed the best yield results. However, the results indicated the opposite regarding α-glucosidase inhibitory compounds. The heterotic effect of functional biochemicals was observed for traits related to genotypes, polyphenol content, antioxidant activity, α-glucosidase inhibitory compounds, and similar properties related to yield and yield components, indicating their use in hybrid chili production.

## 1. Introduction

Chili is generally known as a food ingredient because of its spiciness, the level of which depends on the species [1]. Five common species are cultivated, including *C. annuum* (lowest spiciness) [2] and *C. chinense* (highest spiciness) [3]. Chili spiciness is caused by a secondary metabolite compound called capsaicin and can be quantified using Scoville heat units [4]. Capsaicin is an active component of chili that can irritate and cause a burning sensation in body tissues [5]. Generally, people use chili spiciness in various ways: as a flavor enhancer [6], as a self-protection spray [7], as cosmetic raw materials [8,9], or for the pharmaceutical development of chili patches [10,11,12]. Recent studies have reported that *C. annuum*’s functional secondary metabolites are more beneficial to human health than other species’ [13].

The chili plant’s most commonly used part is its fruit. The chili fruit has various secondary metabolites with high benefits: capsaicin, alkaloids [14], terpenoids [15], steroids [16], saponins [17], vitamin A [18], vitamin C [18], capsanthin [19], zeaxanthin [20], and cryptoxanthin (used as a dye) [20]. Additionally, chili has various macro minerals (iron, potassium, calcium, phosphorus, and niacin), which can be absorbed by the body to maintain human health [21]. In medicine, chili capsaicin is used for its analgesic properties, relieving asthma and skin itchiness. Additionally, recent studies have reported its anticancer, antidiabetic, and antiarthritic activities, as well as its role as a gastric acid secretion stimulant to prevent digestive system infections [5].

Generally, secondary metabolites are produced by plants under environmental stress conditions [22]. However, chili possesses basal antioxidant activity and polyphenol contents [22,23]. Antioxidants counteract free radicals in the body [24], and free radical scavenging helps maintain health and prevent degenerative diseases. One such degenerative disease is diabetes mellitus, which chili can potentially help overcome as a functional food.

The World Health Organization aims to decrease the number of patients with diabetes within the next ten years [25]. Chili consumption can help reach this target through its preventive properties. COVID-19 has changed people’s lifestyles worldwide, making them focus primarily on their diet. A healthy lifestyle includes a diet with foods rich in functional biochemical compounds. Consuming functional biochemical compounds by eating chili is a precautionary measure and helps reduce the side effects of chemical drugs impacting patients [26]. Our previous hybridization research produced a hybrid cultivar with functional biochemical compounds to help overcome this problem [27]. Additionally, chili has α-glucosidase inhibitory (AGI) compounds, which inhibit blood sugar absorption [27,28,29,30].

Chili’s functional compounds can be improved in various ways. Plant cultivation techniques can significantly affect the content of the active compounds in chili through environmental influences [31]. One solution is to create new varieties through genetic improvement. New superior varieties can be developed conventionally or with biotechnology methods. Hybridization is a conventional method that produces hybrids with favorable combined properties (e.g., yield and biochemical content). The obtained hybrid performance information must be supported by combining ability information as the primary consideration in the selection process. Selecting varieties based on the estimated value of the combined abilities for targeted traits will facilitate the development of new chili varieties with high yields and levels of functional biochemical compounds. This study aims to determine the estimated combining ability value of chili genotypes in terms of polyphenols, antioxidant activity, AGI, yield, and yield components in *C. annuum*.

## 2. Materials and Methods

### 2.1. Plant Material

The parent plants used in this study were selected as in previous studies [32,33]. Our previous study reported that the IPB breeding laboratory’s collection of genotypes contained polyphenols, antioxidant activity, and α-glucosidase inhibitors. Five genotypes (IPB367, IPB435, IPB005, IPB374, and IPB074) with distinctive hybridization activity values were selected as parents. IPB367 is an ornamental chili with AGI and high 2,2-diphenyl-1-picrylhydrazyl (DPPH) antioxidant activity [33]. The IPB367 and IPB435 genotypes produce more fruits but with lower weights than other genotypes. Therefore, IPB005, IPB374, and IPB074, which produce a large quantity of chili fruits with greater weights, were selected. The hybridization is expected to produce superior progeny regarding two different traits. Cross-breeding was performed using a diallel design method, where parents and hybrids are used with reciprocity. Hybrids and reciprocals were produced via hybridization in Bogor, Indonesia, at the IPB Alam Sinarsari greenhouse.

### 2.2. Yield Component, Yield, and Biochemical Compounds

The fruits of ten F1 and F1R sample plants were evaluated; 20 fruits were harvested from each experimental unit across 25 treatments. The following growth characteristics were measured: dichotomous height (DH) from the base of the soil to the first dichotomous branch (with a digital ruler); leaf length (LL) from base to tip (with a digital ruler); leaf width (LW) from the left end to the right end (with a digital ruler); and stem diameter (SD), at 5 cm from the ground.

Yield components were also measured: fruit length (FL), using a digital ruler; fruit diameter (FD) by calculating the average diameter of ten fruits in one plant in the third to fifth harvests (with a digital caliper); fruit thickness (FT) by calculating the average thickness of ten fruits in one plant in the third to fifth harvests (with a digital caliper); fruit weight (FW) by calculating the average weight of ten fruits in one plant in the third to fifth harvest (with a digital analytical balance); the number of fruits per plant (NFP) by counting the total fruit number on one plant during the eight harvests; and yield by calculating the total fruit weight of one plant during the eight harvests(with a digital analytical balance). Perfectly ripe red chilies were used for biochemical measurements. The following biochemical compounds were sequentially measured: total phenolic content (TPC) using the gallic acid standard, total flavonoid content (TFC) using the quercetin standard, antioxidant activity of DPPH using the ferric reduction antioxidant power method (FRAP) with Trolox standard, and AGI using the acarbose standard. All measurements were performed via ELISA Reader Spectrophotometry at the following wavelengths: 750, 415, 517, 595, and 410 (following the order cited above). The measured absorbance results were converted using standard charts and analyzed to estimate the combining ability and heterotic effect. The extraction was performed using 3 g of chili powder dissolved in 20 mL of 70% ethanol, and samples were left on an orbital shaker for 48 h. Chili powder was produced by drying fresh fruit until the water content reached 10%. Then, the fruit was ground into powder using a grinder to 20 mesh size. Samples were then filtered and deposited in a vial for 24 h in the refrigerator [34].

### 2.3. Data Statistical Analysis

The randomized complete block design was used for the full diallel analysis of five parents [27]. Chili was cultivated in a greenhouse under a controlled environment, following chili cultivation procedures. General combining ability (GCA), specific combining ability (SCA), and heterotic effect of functional compounds were estimated using the full diallel method following Griffing’s methodology [1956; Method I, Model 1 (fixed effect)]. All performance data were analyzed using “Analysis of Genetics Designs” in R (AGD-R) v. 5.0. (Centro Internacional de Mejoramiento de Maíz y Trigo). The raw data were collected from field and laboratory analysis with three replications. Each replication used ten samples for analysis. The statistical model was defined as follows:

Yij = m + gi + gj + sij + rij + 1/bc ΣΣeijkl

where Yij is the mean value of hybrid and parent genotypes; m is the effect of general means; gi and gj are effects of the GCA related to the i-th and j-th parent, respectively; sij is the effect of SCA for the crossings between the parents of order i and j; rij is the reciprocal effect; and 1/bc ΣΣeijkl is the mean’s value of error.

The critical difference test (CD) was conducted to evaluate differences in GCA between parents based on Griffing method diallel analysis If GCA > CD, the GCA was considered significantly different. The heterosis and heterobeltiosis concerning the mean of the parents was calculated for each character, using the AGD-R program:

CD = √(MSe/p) × t (5% table)

where CD is the critical difference test; MSe is the mean square error, and *p* is the number of parents.

## 3. Results

### 3.1. Analysis of Variance

GCA and SCA estimates were essential components obtained from chili plant breeding activities. Variance analysis in this study significantly affects the GCA mean square for growth characters, yield, yield components, and biochemical content in all observed variables. The SCA mean square also significantly affected all observed variables (Table 1, Table 2 and Table 3).

Sources of reciprocal diversity in all variables observed for growth characteristics, yield, yield components, and biochemical content displayed a significant effect at the 1% level. The significant effect on reciprocity indicated that the F1 and F1 reciprocals tested in this study differed significantly. The significantly different information on the diversity sources in GCA and SCA is taken as the basis for further estimating combining abilities. Thus, the coefficients of variance of diversity in growth characteristics, yield, and yield components were between 1.61 and 5.03% and 2.64 and 6.81% (Table 1 and Table 2). The coefficient of variance of the variable observed for biochemical content was between 1.60 and 3.26% (Table 3).

### 3.2. GCA Analysis

IPB005 was the parent genotype with the highest GCA for several growth characteristics and yield components, including LW, FD, FT, FW, NFP, and yield. The best GCA for the SD, LL, and FL characteristics was displayed by parent IPB374 (Table 4 and Table 5). IPB074 had the best GCA for DH and TPC. Meanwhile, IPB435 displayed the best GCA for TFC and FRAP and IPB367 displayed the best GCA for DPPH and AGI.

The GCA results for yield and yield components varied between parents (Table 5). Chili lines IPB005 and IPB374 had positive GCAs for FL, FT, FW, and yield. Meanwhile, the chili lines IPB367 and IPB435 had negative GCAs for the same characteristics, especially IPB367, which had the highest negative GCA for FL (GCA −2.86) and NFP (GCA −2.49). The IPB435 parent had the highest negative GCA for FD (−3.14), FW (−3.38), and yield (−238.53).

The highest GCA values for five parents were evenly distributed. Each parent displayed advantages in the desired characteristic. The parents IPB005, IPB367, and IPB074 have positive GCAs for DPPH antioxidant activities, while IPB374 and IPB435 exhibited the highest negative GCAs for DPPH antioxidant activities. Only parents IPB005 and IPB435 had a positive GCA for FRAP antioxidant activities (0.56 and 4.62, respectively). IPB435 had positive GCA results for AGI (3.99) and TFC (0.35). Meanwhile, IPB435 had a negative GCA for TPC (−0.80). IPB367 displayed a positive GCA on all biochemical observations, including TPC, TFC, DPPH, and AGI but not the FRAP antioxidant activity, with respective values of 2.18, 0.18, 0.08, 9.86, and −3.07 (Table 6).

### 3.3. SCA Analysis

Parents with positive and negative SCAs in DH and SD (12 and 11 combinations of crosses) exhibited positive SCAs in LL and LW. The highest SCA for DH was displayed by hybrid IPB374 × IPB435 (4.33), followed by hybrids IPB005 × IPB435 (3.67), IPB074 × IPB435 (3.64), and IPB074 × IPB374 (3.44). The IPB435 × IPB374 and IPB435 × IPB367 hybrids had negative SCAs for LL (−0.61 and −0.73) and leaf width (−0.47 and −0.37). Positive SCA values for all growth characteristics were displayed by hybrid IPB367 × IPB005, which also had the highest positive SCA for LL (0.69) (Table 7).

Yield and yield components for SCA estimates included FL, FD, FT, FW, and NFP (Table 8). SCAs for FL were between −1.12 and +1.12. The lowest and highest SCAs for FL were in hybrids IPB005 × IPB435 and IPB005 × IPB374. The three highest SCA values for FD were seen in hybrids IPB074 x IPB005, IPB367 × IPB005, and IPB435 × IPB367 (2.51, 2.49, and 1.74, respectively). Hybrid IPB074 × IPB005 had a negative SCA value (−0.43) for FT.

The FW, NFP, and FW per plant were the main characteristics that determined potential chili productivity. The highest SCA values for FW were observed in hybrids IPB074 × IPB005, IPB374 × IPB005, and IPB005 × IPB367. The highest SCAs for NFP were observed in hybrids IPB435 × IPB374, IPB074 × IPB367, and IPB435 × IPB005. Meanwhile, the highest SCA for FW per plant was for hybrid IPB074 × IPB005, which was recommended for its high potential yield (Table 8).

Eight hybrids had positive SCAs and 12 had negative SCAs for the TPC and AGI characteristics. SCA values for antioxidant activity displayed contradictory results depending on the method used. DPPH had 13 positive SCA values in contrast to FRAP, which had 13 negative SCA values. The hybrid IPB367 × IPB374 had the lowest negative SCA for the three biochemical constituents (TPC: −9.57; TFC: −0.89; FRAP: −12.36). The highest SCAs for AGI and TPC were observed for hybrid IPB074 × IPB374 (7.83 and 4.06). Hybrid IPB435 × IPB367 exhibited the highest positive SCAs for TFC (1.11) and FRAP (8.23). Finally, hybrid IPB367 × IPB374 had the highest positive SCA for DPPH (0.16) (Table 9).

### 3.4. Heterotic and Heterobeltiotic Effects in Chili

The results of heterosis and heterobeltiosis analyses on growth characteristics, yield components, and biochemical content are presented in Table 10, Table 11 and Table 12. The heterotic effect for DH was −20.75–32.95%, whereas the effect of heterobeltiosis was −25.61–24.24%. The highest heterotic and heterobeltiotic effects for this characteristic were observed in hybrids IPB435 × IPB074 and IPB374 × IPB074. Hybrid IPB435 × IPB374 consistently displayed the second-highest heterotic and heterobeltiotic effects. The highest heterotic effect for leaves (LL and LW) was observed in hybrid IPB367 × IPB005; the heterotic and heterobeltiotic effects were 35.98% and 9.36% for LL and 15.46% and −8.94% for LW, respectively. These effects were the highest for SD in hybrid IPB435 × IPB374. The hybrids IPB367 × IPB374 and IPB367 × IPB435 had the overall lowest heterobeltiotic effect (Table 10).

The highest effects for fruit length (HMP, 4.47%; HHP, −1.83%) were displayed by two different hybrids, IPB374 × IPB005 and IPB435 × IPB367. FD and FT resulted in heterotic effect values of −33.84–22.69% and −41.06–5.19%. Meanwhile, the heterobeltiotic effect for these two characteristics was −51.88–−1.79% and −54.48–−6.79%, with the highest values displayed by IPB435 × IPB374 and IPB074 × IPB374. The highest heterotic and heterobeltiotic effects for the three characteristics FW, NFP, and yield were observed in IPB435 × IPB367, IPB435 × IPB374, and IPB005 × IPB374. The highest heterobeltiosis values differed in the FW characteristic for hybrid IPB005 × IPB074, which had the highest value. The heterotic effect for FW per plant was −75.44%–15.20% (Table 11).

The heterotic and heterobeltiotic effects for TPC were −56.09–13.91% and −56.31–4.21%, respectively. The highest and lowest effects for this characteristic were displayed by hybrids IPB435 × IPB074 and IPB367 × IPB374, respectively. The best effects for TFC were observed in hybrid IPB005 × IPB074, followed by the reciprocal hybrid IPB074 × IPB005. Interestingly, the same highest value was observed with the FRAP method. The best effects for DPPH were seen in hybrid IPB074 × IPB435. The heterobeltiotic effect for FRAP was negative for all hybrids. The highest effects for AGI were seen in hybrid IPB005 × IPB074, followed by the reciprocal hybrid IPB074 × IPB374 (Table 12).

## 4. Discussion

Improving chili varieties through hybridization remains a common practice among chili breeders. Hybridization takes advantage of Mendel’s segregation laws, where genetic carriers of superior traits are exchanged between two parents. The first chili breeding milestone is improving productivity, followed by enhancing plant resistance toward pests and diseases. Developing global issues such as COVID-19 pose a new challenge for chili breeders to obtain superior varieties to use as functional foods. This study evaluated five *C. annuum* parents (self-pollinated) and 20 hybrids and their reciprocals regarding the yield and functional biochemical contents, including polyphenols, antioxidants, and AGI compounds.

The performance of hybrids and parents indicates the success of upgrading a particular characteristic. However, at the genetic level, such performance can be predicted through diallel analysis so that plant breeders can select the best parents for further crosses. Diallel analysis can provide information on the estimated effects of the GCA and SCA. This estimation can identify segregated populations with the potential for high genetic variability and superior hybrid combinations [34,35,36,37]. Information on GCA and SCA estimation is crucial and is obtained by diallel analysis using the Griffing method [38]. Genetic studies using this method reveal information on the magnitude of the GCA effect, which can indicate the superiority of the dominant parent regarding its additive effects [39]. Additionally, the SCA effect can also be estimated, demonstrating the superiority of dominant hybrids regarding nonadditive results. This analysis can also indicate the expected behavior of the resulting hybrid population based on the parents’ GCA [40].

Parents with high GCA values can increase the expression of the resulting hybrids. Meanwhile, a low GCA value indicates that the parent does not differ from the average of the entire diallel. A higher GCA value contributes to increasing the expression of the intended trait. A positive or negative GCA value indicates that the parent is superior or inferior, respectively. However, a low GCA is better for some traits, like disease resistance [41], plant height, and canopy width in ornamental plants [42,43]. A less negative value for yield components refers to decreased potential yield [44]. Information on GCA and SCA is used to determine parents and appropriate breeding methods to improve the selected characteristic in plants [45].

GCA estimates are used to determine parents for future research [46]. A parent with a high GCA could also be used for synthetic varieties and hybrid development programs [47]. Variety development based on growth characteristics can use DH, LL, and short LW. In this study, the IPB367 genotype was the best for chili’s ornamental development. In contrast, increased yield and yield components require tall plants, sturdy stems, and broad leaves to maximize photosynthesis and produce large fruits [48].

Variety development programs aiming to increase productivity can rely on parent GCA values (Table 4, Table 5 and Table 6). The highest GCAs for yield were observed for IPB005, IPB074, and IPB374. These three genotypes are also recommended to develop new varieties for large fruits. IPB435 had a high GCA for NFP, in addition to IPB005 and IPB374.

IPB435 had a high combining ability, in contrast to IPB367, for FRAP antioxidants. However, IPB367 had better GCAs than other parents for DPPH and AGI. This line is recommended to be a parent in hybridization to produce hybrids with high DPPH and AGI antioxidants. In line with our previous study, these results suggest that parents with high GCA values for capsaicin could be used to develop spicy *C. annuum* varieties [49].

The SCA effect is a reflection produced by the hybrid based on the GCA value. The hybrid IPB074 × IPB005 had the highest SCA for yield resulting from the two parents with the highest positive GCA. Interestingly, for the same characteristic in this study, a hybrid combination with the second highest positive value (hybrid IPB435 × IPB367) resulted from the two parents with negative GCA values. Additionally, the SCA value of positive yield characteristics could be produced from two parents with negative GCAs in plant height characteristics [50]. The AGI displayed the same trend, with the highest SCA value displayed by IPB074 × IPB374, which was produced from two parents with a negative GCA, including one with the highest negative GCA (IPB074). This is caused by positive genes spreading within the parents and covering the negative genes in the partner parents so they can combine well [51]. A high SCA value indicates better or worse performance than expected, depending on the evaluated characteristics. SCA also estimated the effect of nonadditive and epistatic genes [52].

Diallel analysis can also predict heterotic and heterobeltiotic effects. The heterotic effect indicates that the hybrid performance is better than the means of parents, while the heterobeltiotic effect suggests that the hybrid performance is better than the best parents [53,54,55]. Parents whose poor performance makes them not easy to use can be combined to become hybrids with the best heterotic and heterobeltiotic effects. The highest heterotic effects in leaf characteristics (LL and LW) were seen in IPB367 × IPB005, which also had the highest positive SCA for LL. This result indicates that parents with positive and negative GCAs produce hybrids with the highest SCAs and heterotic effects regarding leaves. A high SCA also results in a high heterotic effect [56]. Meanwhile, heterotic and heterobeltiotic effects for yield were displayed by different parent combinations (IPB005 × IPB374 and IPB005 × IPB074) with the same female parent (IPB005). This result aligns with our previous research [49] evaluating chili production for use as a spice with good heterotic effects in the hybrids evaluated.

The highest heterotic and heterobeltiotic effects for TFC, DPPH, and AGI were displayed by IPB005 × IPB074, IPB074 × IPB114, and IPB374 × IPB074. Interestingly, this study’s heterobeltiosis effect for antioxidant FRAP was negative for all hybrids. The heterotic effect is formed by crossing two parents with different gene frequencies [57], which explains that the self-pollination genotype has a zero heterotic effect. Several studies have demonstrated the benefits of using chili hybrids by exploiting the heterotic phenomenon [58].

Commercial breeding programs are required to produce new varieties based on market demand. New varieties are obtained from hybridization of two or more genotypes as parents to create new hybrids. However, producing new varieties requires GCA and SCA information as fundamental information to determine the direction of variety development. The information generated from this study can be further implied based on market demand. Genotypes with high GCA provide potential hope for use in further planting. The limitations in this study can be improved in further studies such as planting outside the greenhouse with more genotypes as parents so that it can recommend other genotypes with a broader scope for precision breeding programs.

## 5. Conclusions

IPB005, IPB074, IPB374, IPB367, and IPB435 had high affinity potential and advantages as parents based on the observed characteristics. They can form a superior segregated population determined by the direction of the breeding program. The hybrid IPB374 × IPB074 is the best combination considering AGI production; it can be developed for the pharmaceutical industry. Additionally, the hybrid IPB005 × IPB074 displayed the best yield. All hybrids observed had a heterotic effect; they could be developed for further research. The GCA and SCA values produced in this study can help breeders develop hybrid chili plants. Moreover, the heterotic effect produced in this study confirmed that the hybrid means are higher than those of individual parents.

## Figures and Tables

**Table 1:** ANOVA of chili growth characteristics for diallel mating design.

Sources	df	Mean Square			
		DH	LL	LW	SD
GCA	4	219.98 **	10.25 **	1.71 **	6.85 **
SCA	10	15.91 **	0.95 **	0.25 **	3.05 **
Reciprocal	10	8.91 **	1.01 **	0.22 **	8.35 **
Error	48	0.68	0.03	0.01	0.01
Coefficient of Variance (%)		5.03	3.20	4.86	1.61

df, degree of freedom; GCA, general combining ability; SCA, specific combining ability; DH, dichotomous height; LL, leaf length; LW, leaf width; SD, stem diameter; ** significant at *p* ≤ 0.01.

**Table 2:** ANOVA of chili yield and yield components.

Sources	df	Mean Square					
		FL	FD	FT	FW	NFP	Yield
GCA	4	55.76 **	85.93 **	0.48 **	88.41 **	39.04 **	445617.7 **
SCA	10	0.94 **	8.05 **	0.14 **	9.63 **	796.63 **	35735.5 **
Reciprocal	10	0.67 **	0.93 **	0.06 **	0.29 **	3.79 ns	1008.59 **
Error	48	0.03	0.03	0.002	0.05	3.61	307.77
Coefficient of Variance (%)		4.03	2.64	5.03	6.17	4.71	6.81

df, degree of freedom; GCA, general combining ability; SCA, specific combining ability; FL, fruit length; FD, fruit diameter; FT, fruit thickness; FW, fruit weight; NFP, fruit number per plant; ** significant at *p* ≤ 0.01; ns, not significant.

**Table 3:** ANOVA of chili biochemical compounds.

Sources	df	Mean Square				
		TPC	TFC	DPPH	FRAP	AGI
GCA	4	100.18 **	0.66 **	0.07 **	86.75 **	500.27 **
SCA	10	129.29 **	1.31 **	0.04 **	150.56 **	40.73 **
Reciprocal	10	1.71 **	0.01 **	0.002 **	3.39 **	0.81 **
Error	48	0.26	0.002	0.001	0.42	0.38
Coefficient of Variance (%)		3.16	3.20	1.19	3.26	1.60

df, degree of freedom; GCA, general combining ability; SCA, specific combining ability; TPC, total phenolic content; TFC, total flavonoid content; DPPH, antioxidant activities; FRAP, antioxidant activities; AGI, α-glucosidase inhibitor; ** significant at *p* ≤ 0.01.

**Table 4:** GCAs for chili growth characters.

Genotypes	DH	LL	LW	SD
IPB005	−1.27 ^d^	0.56 ^b^	0.47 ^a^	0.15 ^b^
IPB374	3.13 ^b^	0.77 ^a^	0.32 ^b^	1.25 ^a^
IPB367	−6.94 ^e^	−1.75 ^e^	−0.57 ^e^	0.06 ^b^
IPB435	−0.24 ^c^	0.37 ^c^	−0.21 ^d^	−0.51 ^c^
IPB074	5.33 ^a^	0.06 ^d^	−0.01 ^c^	−0.95 ^d^
Critical difference	0.79	0.16	0.10	0.10

DH, dichotomous height; LL, leaf length; LW, leaf width; SD, stem diameter. Numbers followed by the same letter in the same column are not significantly different according to the critical difference test at 5%.

**Table 5:** GCAs for chili yield and yield components.

Genotypes	FL	FD	FT	FW	NFP	Yield
IPB005	0.88 ^c^	4.47 ^a^	0.28 ^a^	3.17 ^a^	2.14 ^a^	239.24 ^a^
IPB374	2.57 ^a^	−0.39 ^c^	0.16 ^b^	1.10 ^c^	0.87 ^a^	69.99 ^c^
IPB367	−2.86 ^e^	−1.87 ^d^	−0.09 ^c^	−2.93 ^d^	−2.49 ^b^	−203.75 ^d^
IPB435	−2.11 ^d^	−3.14 ^e^	−0.27 ^d^	−3.38 ^e^	1.14 ^a^	−238.53 ^e^
IPB074	1.53 ^b^	0.93 ^b^	−0.08 ^c^	2.04 ^b^	−1.66 ^b^	133.05 ^b^
Critical difference	0.16	0.16	0.04	0.21	1.81	16.71

FL, fruit length; FD, fruit diameter; FT, fruit thickness; FW, fruit weight; NFP, number of fruits per plant. Numbers followed by the same letter in the same column are not significantly different according to the critical difference test at 5%.

**Table 6:** GCAs for chili biochemical contents.

Genotype	TPC	TFC	DPPH	FRAP	AGI
IPB005	−3.08 ^d^	−0.14 ^c^	0.04 ^b^	0.56 ^b^	−3.88 ^d^
IPB374	−2.59 ^d^	−0.26 ^d^	−0.09 ^c^	−0.22 ^c^	−1.65 ^c^
IPB367	2.18 ^b^	0.18 ^b^	0.08 ^a^	−3.07 ^e^	9.86 ^a^
IPB435	−0.80 ^c^	0.35 ^a^	−0.09 ^c^	4.62 ^a^	3.99 ^b^
IPB074	4.30 ^a^	−0.13 ^c^	0.05 ^ab^	−1.89 ^d^	−8.32 ^e^
Critical difference	0.49	0.04	0.03	0.62	0.59

TPC, total phenolic content; TFC, total flavonoid content; DPPH, antioxidant activities; FRAP antioxidant activities; AGI, α-glucosidase inhibitor. Numbers followed by the same letter in the same column are not significantly different according to the critical difference test at 5%.

**Table 7:** SCAs for chili growth characteristics.

Chili Hybrid			DH	LL	LW	SD
IPB005	×	IPB374	−0.33	0.33	0.68	−1.24
IPB005	×	IPB367	1.5	0.4	0.001	−3.62
IPB005	×	IPB435	3.67	0.67	−0.12	0.47
IPB005	×	IPB074	−1.00	0.37	0.4	−1.41
IPB374	×	IPB005	1.37	0.68	0.43	−0.78
IPB374	×	IPB367	−1.83	−0.08	0.001	−3.89
IPB374	×	IPB435	4.33	−1.42	−0.42	2.51
IPB374	×	IPB074	−0.67	−1.18	−0.48	1.64
IPB367	×	IPB005	1.61	0.69	0.13	1.67
IPB367	×	IPB374	−0.46	0.004	0.28	−0.34
IPB367	×	IPB435	−0.17	0.55	0.2	0.62
IPB367	×	IPB074	−1.33	0.13	−0.08	0.59
IPB435	×	IPB005	−2.26	0.61	0.181	−0.69
IPB435	×	IPB374	0.67	−0.61	−0.47	1.37
IPB435	×	IPB367	0.24	−0.73	−0.37	−2.03
IPB435	×	IPB074	−1.83	0.68	0.02	−0.25
IPB074	×	IPB005	−2.16	−0.05	−0.202	0.62
IPB074	×	IPB374	3.44	−0.24	−0.27	2.66
IPB074	×	IPB367	1.17	0.42	0.15	−0.06
IPB074	×	IPB435	3.64	−0.04	−0.02	2.46

DH, dichotomous height; LL, leaf length; LW, leaf width; SD, stem diameter.

**Table 8:** SCAs for chili yield and yield components.

Chili Hybrids			FL	FD	FT	FW	NFP	Yield
IPB005	×	IPB374	1.12	0.61	−0.26	0.75	0.5	−22.82
IPB005	×	IPB367	0.65	0.15	0.05	0.81	0.83	16.51
IPB005	×	IPB435	−1.12	−0.72	−0.34	−0.04	−0.17	−7.38
IPB005	×	IPB074	−0.28	−0.38	−0.01	−0.19	−3.00	−54.32
IPB374	×	IPB005	0.4	0.66	−0.08	1.78	−5.27	100.69
IPB374	×	IPB367	0.23	−0.67	0.32	−0.37	0.001	−0.31
IPB374	×	IPB435	0.12	0.12	0.003	−0.11	0.5	−1.67
IPB374	×	IPB074	0.03	−1.18	0.11	−0.19	−1.5	−34.32
IPB367	×	IPB005	−0.81	2.49	−0.04	−2.34	8.76	−115.88
IPB367	×	IPB374	−0.38	−0.24	−0.19	−0.76	−18.47	−61.09
IPB367	×	IPB435	0.1	0.28	0.013	0.05	−1.33	2.06
IPB367	×	IPB074	0.001	0.09	−0.04	0.1	−0.17	7.695
IPB435	×	IPB005	−0.56	−2.81	−0.03	−3.07	11.46	−102.67
IPB435	×	IPB374	0.02	1.39	−0.27	−0.79	27.39	17.53
IPB435	×	IPB367	0.46	1.74	0.08	2.77	−28.07	104.03
IPB435	×	IPB074	−0.52	1.28	0.09	−0.09	2.17	−0.61
IPB074	×	IPB005	0.01	2.51	−0.43	2.09	0.76	135.85
IPB074	×	IPB374	−0.99	−0.75	0.26	−1.84	5.19	−90.17
IPB074	×	IPB367	−0.31	−2.13	−0.19	−1.53	16.56	−71.09
IPB074	×	IPB435	0.19	−0.82	0.12	−1.16	−30.74	−206.33

FL, fruit length; FD, fruit diameter; FT, fruit thickness; FW, fruit weight; NFP, number of fruits per plant.

**Table 9:** SCAs for chili biochemical compounds.

Chili Hybrids			TPC	TFC	DPPH	FRAP	AGI
IPB005	×	IPB374	0.17	−0.01	0.001	−0.61	−0.22
IPB005	×	IPB367	−0.28	0.05	−0.03	−0.67	−0.5
IPB005	×	IPB435	−0.42	0.06	0.04	−0.46	0.24
IPB005	×	IPB074	−0.69	−0.03	0.04	0.49	−0.88
IPB374	×	IPB005	−3.26	0.08	−0.1	−3.53	−3.09
IPB374	×	IPB367	−1.01	−0.01	−0.01	0.52	−0.15
IPB374	×	IPB435	0.15	0.14	0.04	3.24	−1.03
IPB374	×	IPB074	−1.12	0.07	0.04	−1.12	−0.2
IPB367	×	IPB005	−6.06	−0.73	0.11	−0.92	0.68
IPB367	×	IPB374	−9.57	−0.89	0.16	−12.36	4.4
IPB367	×	IPB435	−0.35	0.02	0.03	0.88	−0.61
IPB367	×	IPB074	0.52	0.02	0.02	1.39	0.76
IPB435	×	IPB005	−7.39	−0.48	0.07	−6.58	2.99
IPB435	×	IPB374	−7.44	−0.47	−0.014	−1.93	−2.8
IPB435	×	IPB367	7.72	1.11	−0.28	8.23	−0.52
IPB435	×	IPB074	−2.26	−0.03	0.03	−0.97	0.91
IPB074	×	IPB005	2.99	0.76	−0.02	5.96	−6.68
IPB074	×	IPB374	4.06	0.26	0.01	−0.27	7.83
IPB074	×	IPB367	1.65	−0.85	−0.03	−10.17	0.23
IPB074	×	IPB435	0.58	−0.64	0.2	−2.89	−0.004

TPC, total phenolic content; TFC, total flavonoid content; DPPH, antioxidant activities DPPH method; FRAP, antioxidant activities FRAP method; AGI, α-glucosidase inhibitory.

**Table 10:** Heterotic and heterobeltiotic effects for chili growth characteristics.

Chili Hybrids			TD		PD		LD		DBT	
			HMP	HHP	HMP	HHP	HMP	HHP	HMP	HHP
			………………………………………… (%) ……………………………………………….							
IPB005	×	IPB374	12.28	7.87	13.55	−0.32	0	−3.05	32.79	31.77
IPB005	×	IPB367	3.39	−25.61	23.28	−0.85	15.46	−8.94	68.68	54.86
IPB005	×	IPB435	−20.75	−23.17	5.93	−6.23	5.79	4.07	−3.2	−14.22
IPB005	×	IPB074	3.87	−5.05	7.26	1.92	−12.3	−13.01	30.44	24.99
IPB374	×	IPB005	9.94	5.62	20.88	6.11	32.28	28.24	−2.13	−2.88
IPB374	×	IPB367	24.8	−12.36	2.64	−25.08	10.89	−14.5	59.42	47.39
IPB374	×	IPB435	0.00	−6.74	3.25	2.25	−9.6	−13.74	13.71	0.09
IPB374	×	IPB074	30.85	24.24	9.44	0.64	0.79	−3.05	30.66	24.28
IPB367	×	IPB005	18.64	−14.63	35.98	9.36	15.46	−8.94	−24.63	−30.81
IPB367	×	IPB374	7.2	−24.72	0.44	−26.69	10.89	−14.5	−40.37	−44.87
IPB367	×	IPB435	15.04	−15.58	−19.64	−40.98	−25.26	−40.34	−35.54	−46.96
IPB367	×	IPB074	30.37	−11.11	7.92	−16.48	5.21	−16.53	−18.87	−28.36
IPB435	×	IPB005	6.92	3.66	20.74	6.89	0	−1.63	11.67	−1.04
IPB435	×	IPB374	31.33	22.47	−24.35	−25.08	−29.6	−32.82	93.29	70.14
IPB435	×	IPB367	13.27	−16.88	−4.91	−30.16	−12.63	−30.25	−17.69	−32.27
IPB435	×	IPB074	32.95	18.18	−11.31	−17.7	−13.33	−14.05	48.25	36.6
IPB074	×	IPB005	−2.76	−11.11	16.13	10.34	7.38	6.5	−11.36	−15.07
IPB074	×	IPB374	26.6	20.2	−15.38	−22.19	−22.22	−25.19	78.63	69.91
IPB074	×	IPB367	18.52	−19.19	11.88	−13.41	0	−20.66	−3.18	−14.49
IPB074	×	IPB435	20.45	7.07	3.18	−4.26	−12.5	−13.22	39.97	28.97

DH, dichotomous height; LL, leaf length; LW, leaf width; SD, stem diameter; HMP, heterotic mid parent (heterotic effect); HHP, heterotic high parent (heterobeltiotic effect).

**Table 11:** Heterotic and heterobeltiotic effects for chili yield and yield components.

Chili Hybrids			FL		FD		FT		FW		NFP		Yield	
			HMP	HHP	HMP	HHP	HMP	HHP	HMP	HHP	HMP	HHP	HMP	HHP
			…….………………………………………… (%) …………………………………………………											
IPB005	×	IPB374	−13.59	−23.93	−2.94	−30.42	−10.14	−19.01	−4.54	−18.4	10.74	6.91	15.2	−3.06
IPB005	×	IPB367	−35.24	−57.63	−31.14	−48.79	−23.78	−37.53	−60	−76.95	7.16	−10.08	−35.86	−61.46
IPB005	×	IPB435	1.86	−31.78	−23.67	−51.88	−1.53	−29.78	−63.11	−79.4	12.64	−7.97	−33.85	−61.21
IPB005	×	IPB074	−6.57	−12.09	6.46	−12.29	−37.37	−49.27	2.27	−2.71	11.28	−0.89	9	6.85
IPB374	×	IPB005	4.47	−8.03	4.48	−25.1	−31.09	−37.89	8.02	−7.67	12.4	8.51	9.22	−8.09
IPB374	×	IPB367	−18.62	−49.76	−5.74	−10.94	−41.06	−47.06	−35.25	−60.67	−33.18	−42.25	−37.22	−59.34
IPB374	×	IPB435	−5.84	−40.76	19.78	−4.11	−27.49	−44.49	−45.34	−68.19	27.59	7.25	−23.46	−52.04
IPB374	×	IPB074	−15.78	−21.56	2.93	−14.08	−6.04	−16.74	−32.51	−39.73	10.19	1.34	−24.08	−37.13
IPB367	×	IPB005	−16.67	−45.48	−29.39	−47.49	−19.65	−34.14	−39.96	−65.4	9.47	−8.14	−29.8	−57.82
IPB367	×	IPB374	−13.24	−46.45	−18.5	−22.99	−8.48	−17.8	−47.4	−68.05	−33.18	−42.25	−37.37	−59.44
IPB367	×	IPB435	−2.88	−7.34	−4.18	−26.47	−10.23	−25.19	35.16	19.22	−53.18	−54.71	−37.55	−42.01
IPB367	×	IPB074	−17.93	−47.8	−33.84	−42.03	−25.38	−26.52	−51.21	−71.49	2.49	−4.26	−47.32	−68.56
IPB435	×	IPB005	−29.3	−52.65	−33.73	−58.23	−36.16	−54.48	−64.04	−79.93	12.2	−8.33	−36.62	−62.83
IPB435	×	IPB374	−3.2	−39.1	22.69	−1.79	−27.09	−44.19	−49.03	−70.34	28.88	8.33	−24.32	−52.58
IPB435	×	IPB367	2.88	−1.83	2.3	−21.49	−8.41	−23.67	40.48	23.91	−56.18	−57.61	−35.12	−39.76
IPB435	×	IPB074	2.75	−33.24	−28.45	−49.83	−10.42	−24.41	−47.85	−70.56	−56.4	−60.51	−75.44	−85.68
IPB074	×	IPB005	−11.53	−16.76	2.47	−15.58	−37.97	−49.76	−0.61	−5.45	2.26	−8.93	−2.74	−4.66
IPB074	×	IPB374	−15.27	−21.09	−16.15	−30	5.19	−6.79	−35.96	−42.81	5.83	−2.68	−32.86	−44.4
IPB074	×	IPB367	−17.93	−47.8	−32.48	−40.83	−30.38	−31.44	−48.41	−69.85	2.07	−4.65	−44.59	−66.93
IPB074	×	IPB435	−10.36	−41.76	−3.72	−32.48	2.55	−13.48	−50.47	−72.04	−51.2	−55.8	−75.67	−85.81

FL, fruit length; FD, fruit diameter; FT, fruit thickness; FW, fruit weight; NFP, number of fruits per plant; HMP, heterotic mid parent (heterotic effect); HHP, heterotic high parent (heterobeltiotic effect).

**Table 12:** Heterotic and heterobeltiotic effects for chili biochemical compounds.

Chili Hybrids			TPC		TFC		DPPH		FRAP		AGI	
			HMP	HHP	HMP	HHP	HMP	HHP	HMP	HHP	HMP	HHP
			…….………………………………………… (%) ………………………………………………									
IPB005	×	IPB374	−49.24	−51.48	−22.9	−28.41	−2.04	−9.11	−31.27	−38.81	−4.52	−10.41
IPB005	×	IPB367	−42.47	−44.75	−50.94	−60.81	8.38	2.98	−24.68	−27.14	0.71	−9.57
IPB005	×	IPB435	−49.85	−51.7	−31.8	−42.38	3.32	−1.36	−23.39	−28.44	−0.64	−7.29
IPB005	×	IPB074	4.63	−7.49	14.91	12.03	3.1	1.36	−1.92	−5.06	−14.45	−25.27
IPB374	×	IPB005	−48.32	−50.6	−23.68	−29.13	−2.1	−9.17	−33.88	−41.14	−5.24	−11.09
IPB374	×	IPB367	−50.95	−51.19	−60.21	−66.24	10.02	−2.61	−61.68	−64.85	14.69	−2.65
IPB374	×	IPB435	−52.62	−56.31	−42.45	−48.06	−2.25	−5.14	−31.88	−35.33	1.91	−10.35
IPB374	×	IPB074	5.16	−10.58	−20.55	−24.43	4.71	−1.29	−26.33	−36.26	22.68	13.62
IPB367	×	IPB005	−43.96	−46.18	−47.62	−58.16	4.85	−0.37	−27.84	−30.2	−0.64	−10.79
IPB367	×	IPB374	−56.09	−56.31	−60.51	−66.49	8.94	−3.57	−59.53	−62.87	14.24	−3.04
IPB367	×	IPB435	4.65	−3.05	4.73	−2.27	−18.86	−26.21	−3.95	−7.37	3	−1.19
IPB367	×	IPB074	7.96	−7.83	−54.06	−62.58	0.34	−6.18	−55.98	−58.73	3.96	−17.12
IPB435	×	IPB005	−52.3	−54.06	−28.18	−39.32	7.84	2.96	−25.47	−30.38	0.04	−6.65
IPB435	×	IPB374	−51.78	−55.53	−33.41	−39.91	2.11	−0.91	−18.79	−22.89	−1.23	−13.12
IPB435	×	IPB367	2.68	−4.87	5.41	−1.64	−15.93	−23.54	−0.07	−3.62	1.42	−2.7
IPB435	×	IPB074	13.91	4.21	−35.44	−44.28	13.94	10.59	−16.9	−24.69	−0.62	−18.16
IPB074	×	IPB005	0.27	−11.34	12.86	10.03	7.97	6.15	0.54	−2.68	−17.57	−27.99
IPB074	×	IPB374	−1.61	−16.33	−15.03	−19.18	8.99	2.76	−31.3	−40.56	21.92	12.91
IPB074	×	IPB367	11.13	−5.12	−52.66	−61.44	2.34	−4.31	−49.15	−52.33	6.29	−15.26
IPB074	×	IPB435	−1.04	−9.47	−37.46	−46.02	17.73	14.27	−21.47	−28.83	2.34	−15.72

TPC, total phenolic content; TFC, total flavonoid content; DPPH, antioxidant activities DPPH method; FRAP, antioxidant activities FRAP method; AGI, α-glucosidase inhibitor; HMP, heterotic mid parent (heterotic effect); HHP, heterotic high parent (heterobeltiotic effect).

## Data Availability

The data can be sent by request to the corresponding author’s email.
